# Supplementary material for: Gene-Activated Matrix with Self-Assembly Anionic Nano-Device Containing Plasmid DNAs for Rat Cranial Bone Augmentation
Source: Materials (Basel). 2021 Nov 22;14(22):7097. doi: 10.3390/ma14227097 (PMC8621468; doi:10.3390/ma14227097)
Supplement: Supplementary file 1 [file materials-14-07097-s001.zip › materials-1434367-supplementary.pdf]

materials

Article

Gene-Activated Matrix with Self-Assembly Anionic Nano-Device Containing Plasmid DNAs for Rat Cranial Bone Augmentation

Masahito Hara [1,2], Yoshinori Sumita [2,*], Yukinobu Kodama [3], Mayumi Iwatake [2], Hideyuki Yamamoto [1,2], Rena Shido [1], Shun Narahara [1], Takunori Ogaeri [2], Hitoshi Sasaki [3] and Izumi Asahina [1]

[1] Department of Regenerative Oral Surgery, Unit of Translational Medicine, Nagasaki University Graduate School of Biomedical Science, 1-7-1 Sakamoto, 852-8588 Nagasaki, Japan; bb55317205@ms.nagasaki-u.ac.jp (M.H.); yamamotohideyuki@nagasaki-u.ac.jp (H.Y.); r-shido@nagasaki-u.ac.jp (R.S.); narashun@nagasaki-u.ac.jp (S.N.); asahina@nagasaki-u.ac.jp (I.A.)
[2] Basic & Translational Research Center for Hard Tissue Disease, Nagasaki University Graduate School of Biomedical Sciences, 1-7-1 Sakamoto, 852-8588 Nagasaki, Japan; iwatake@nagasaki-u.ac.jp (M.I.); ogaeri@nagasaki-u.ac.jp (T.O.)
[3] Department of Hospital Pharmacy, Nagasaki University Hospital, 1-7-1 Sakamoto, 852-8501 Nagasaki, Japan; y-kodama@nagasaki-u.ac.jp (Y.K.); sasaki@nagasaki-u.ac.jp (H.S.)
* Correspondence: y-sumita@nagasaki-u.ac.jp; Tel.: +81-95-819-7706

Citation: Hara, M.; Sumita, Y.; Kodama, Y.; Iwatake, M.; Yamamoto, H.; Shido, R.; Narahara, S.; Ogaeri, T.; Sasaki, H.; Asahina, I. Gene-Activated Matrix with Self-Assembly Anionic Nano-Device Containing Plasmid DNAs for Rat Cranial Bone Augmentation. *Materials* **2021**, *14*, 7097. https://doi.org/10.3390/ma14227097

Academic Editor: Domenico Dalessandri

Received: 8 October 2021
Accepted: 15 November 2021
Published: 22 November 2021

Publisher's Note: MDPI stays neutral with regard to jurisdictional claims in published maps and institutional affiliations.

Copyright: © 2021 by the authors. Licensee MDPI, Basel, Switzerland. This article is an open access article distributed under the terms and conditions of the Creative Commons Attribution (CC BY) license (http://creativecommons.org/licenses/by/4.0/).

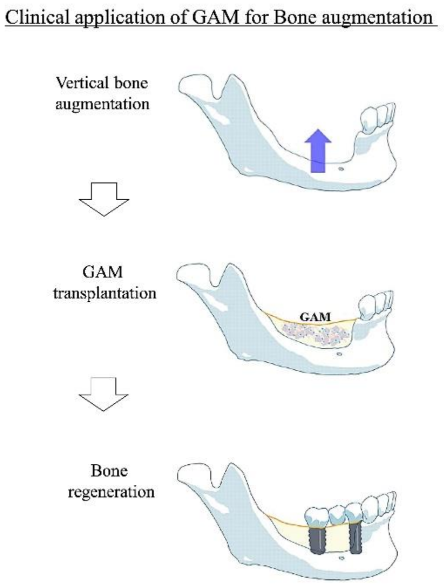

Figure S1. Schematic diagram of the clinical application of GAM for bone augmentation.
